# Life in stop motion: a review of akinetopsia

**DOI:** 10.1186/s13023-025-03781-6

**Published:** 2025-07-02

**Authors:** Salma Mowafi, Rana Khashana, May Bakr

**Affiliations:** 1 Institute of Global Health & Human Ecology, School of Sciences & Engineering, The American University in Cairo, Cairo, Egypt; 2 Department of Psychology, School of Humanities & Social Sciences, The American University in Cairo, Cairo, Egypt

**Keywords:** Visual perception, Akinetopsia, Cortical disorders, Motion blindness, Visual pathways

## Abstract

Akinetopsia is a rare visual cortical disorder in which patients lose the ability to perceive motion. Visual cortical disorders are often misdiagnosed by most clinicians because they misinterpret the cause of visual dysfunction. Since akinetopsia was first described in 1911, only a handful of cases have been studied. Recent cases have demonstrated that it is not necessarily attributed only to vascular causes and neurodegenerative diseases but can also be induced through transcranial magnetic stimulation, and certain medications. This paper aims to review the etiology of akinetopsia in recent studies and provide a more holistic understanding of the disorder and its impact on patients’ lives.

## Introduction

The perception of motion is a fundamental aspect of our daily visual experience. It allows us to navigate our environment, interact with moving objects, and interpret dynamic scenes. However, our understanding of motion perception becomes profoundly complex and intriguing when motion perception is disrupted. Akinetopsia, also referred to as motion blindness, is a rare higher visual processing disorder in which patients lose the ability to perceive motion despite intact vision and the ability to perceive stationary objects [1]. Patients with akinetopsia report difficulties following moving objects or people, as if things “jump” from one place to another or appear as a sequence of static pictures [2, 3]. Everyday tasks such as pouring water or driving are challenging [4, 5]. The first description of akinetopsic symptoms was reported in 1911 by Pötzl and Redlich after their patient sustained a bilateral occipital injury and lost the ability to track moving objects. George Riddoch provided a further description of his patients who sustained gunshot wounds to the primary visual area, V1 [6]. Although the patients were blinded, they were still able to perceive conscious visual motion. This led to the deduction that movement perception is a visual processing function separate from the primary visual cortex. This was later endorsed by Gordon Holmes [7], who described a patient who would perceive the movement of an object but could not identify the object itself or its characteristics, color, or form.

Patient LM [8] and patient AF [9] are two cases of akinetopsia that have been extensively reported in the literature. Patient LM suffered bilateral damage to area V5 of the cortex and could only distinguish between stationery and moving objects at the edge of their unimpaired visual fields. The damage was localized to the lateral temporooccipital cortex and the underlying white matter, and the selective nature of the patient’s visual impairment supported the idea that motion perception is a distinct visual function that depends on neural mechanisms beyond the primary visual cortex [8]. However, the patient’s ability to perceive movement in the central portion of their visual field was preserved if the target velocity did not exceed 10 degrees per second (deg/s). Interestingly, the patient’s perception of movement in response to acoustic and tactile stimuli was not affected. A follow-up on patient LM revealed no change in the deficits experienced, indicating that the damage was irreversible [10].

Patient AF had sustained similar injuries to patient LM: bilateral lesions involving the parietal-temporal-occipital cortex. The patient’s ability to detect coherent motion and discriminate speed became poor following this injury [9]. More recent cases of akinetopsia demonstrate that the ability to perceive motion is a distinct function that relies on neuronal mechanisms beyond those of the primary visual cortex, specifically the MT/V5 area [3, 11–14]. Patients with akinetopsia report that pictures in motion “jump” and that they find it challenging to perform simple tasks such as pouring water [4] and becoming unable to locate moving targets [3]. Heutink and colleagues [15] described the case of patient TD, who had difficulty perceiving the direction of movement at speeds above 9 deg, in agreement with Zihl et al. [8]. The study suggested that the occipitoparietal region’s middle temporal area (MT/V5) is essential for processing high-speed visual motion but not for processing low-speed visual motion. Interestingly, TD always reported the opposite direction of the actual movement at a speed of 24 deg/s, suggesting a form of the continuous wagon-wheel illusion. This illusion also suggests that there could be other brain regions that operate at different sampling rates than area V5 [15].

By tracing the literature on akinetopsia, examining in-depth case studies, and exploring the varying causes of this rare condition, we can gain broader insights into the normal processes of motion perception and the complex neuronal mechanisms that underpin this critical visual function. Through this comprehensive exploration, we aim to enhance the understanding of akinetopsia and contribute to the broader knowledge of visual perception and its neuronal underpinnings. We hope that these insights will pave the way for future research, potentially leading to novel treatment strategies and improved quality of life for individuals affected by this rare disorder.

## Methodology

This literature review employed a structured search strategy to synthesize current evidence on akinetopsia. The search was conducted across multiple databases, including PubMed, Scopus, and Google Scholar. Searches were conducted using a detailed query combining terms related to akinetopsia (e.g., “akinetopsia,” “visual disorder,” “motion blindness”). The search strategy incorporated Boolean logic and database-specific indexing.

### Pathophysiology of the disease

#### Normal visual perception

**Fig. 1:**
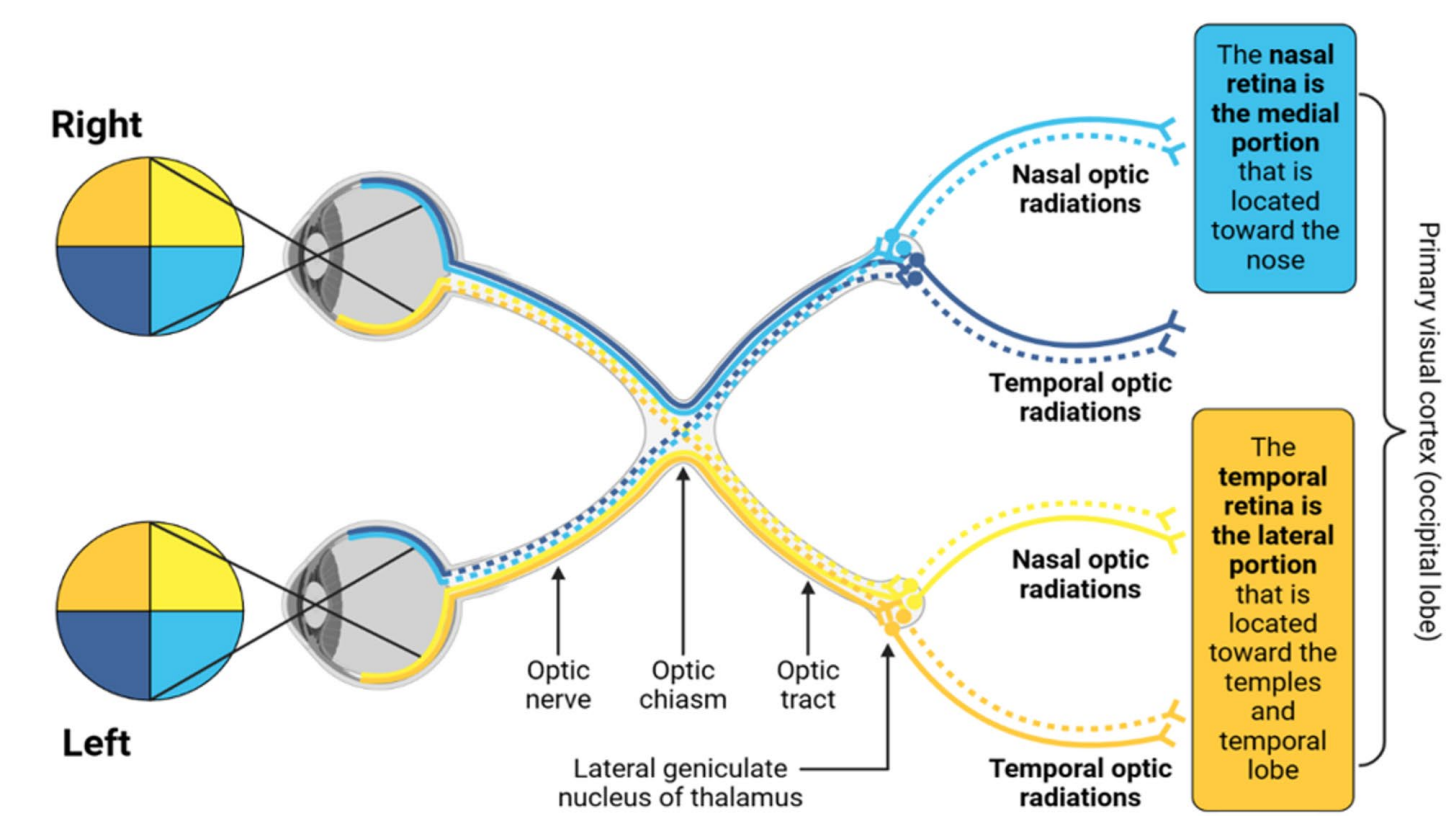
Human visual pathway. The optic tract synapses with the lateral geniculate nucleus of the thalamus. From there, axons project to the primary visual cortex, also called area V1, located in the occipital lobe. Figure created by Biorender.com

The field of psychology defines visual perception as the ability of an individual to interpret the light stimuli that enters their eyes and convert them into information that aids recognition and action. The physical and processing elements that form this assimilation of information from the surroundings are collectively called the visual system. The human visual system is sensitive primarily to light patterns rather than the absolute magnitude of light energy. The eye does not operate as a photometer. Instead, it detects spatial, temporal, and spectral patterns. The pattern of processing a visual stimulus is demonstrated as an intricate system of neuronal interconnections starting from the optic nerve and ending in the visual cortex. Figure 1 illustrates the human visual pathway starting from the retina to the primary visual cortex.

### Role of visual cortices in motion perception

The occipital lobe in the brain houses the visual cortex, which is responsible for receiving and processing visual information from the retina. The visual cortex is divided into five areas, V1 to V5. Visual information is processed in the lateral geniculate nucleus (LGN) of the thalamus before being relayed to cortical area V1 for further processing. This area is located around the calcarine sulcus (Brodmann 17-medial surface of the occipital lobe responsible for spatial recognition and motion filtering). Each hemisphere of the brain has a visual cortex, which receives information from the opposite visual field. This means that the right cortical areas process information from the left eye, and the left cortical areas process information from the right eye. The primary function of the visual cortex is to receive and integrate visual information that is then sent to other parts of the brain for further analysis and use. This specialized process enables the brain to recognize objects and patterns quickly and effortlessly [16].

**Fig. 2:**
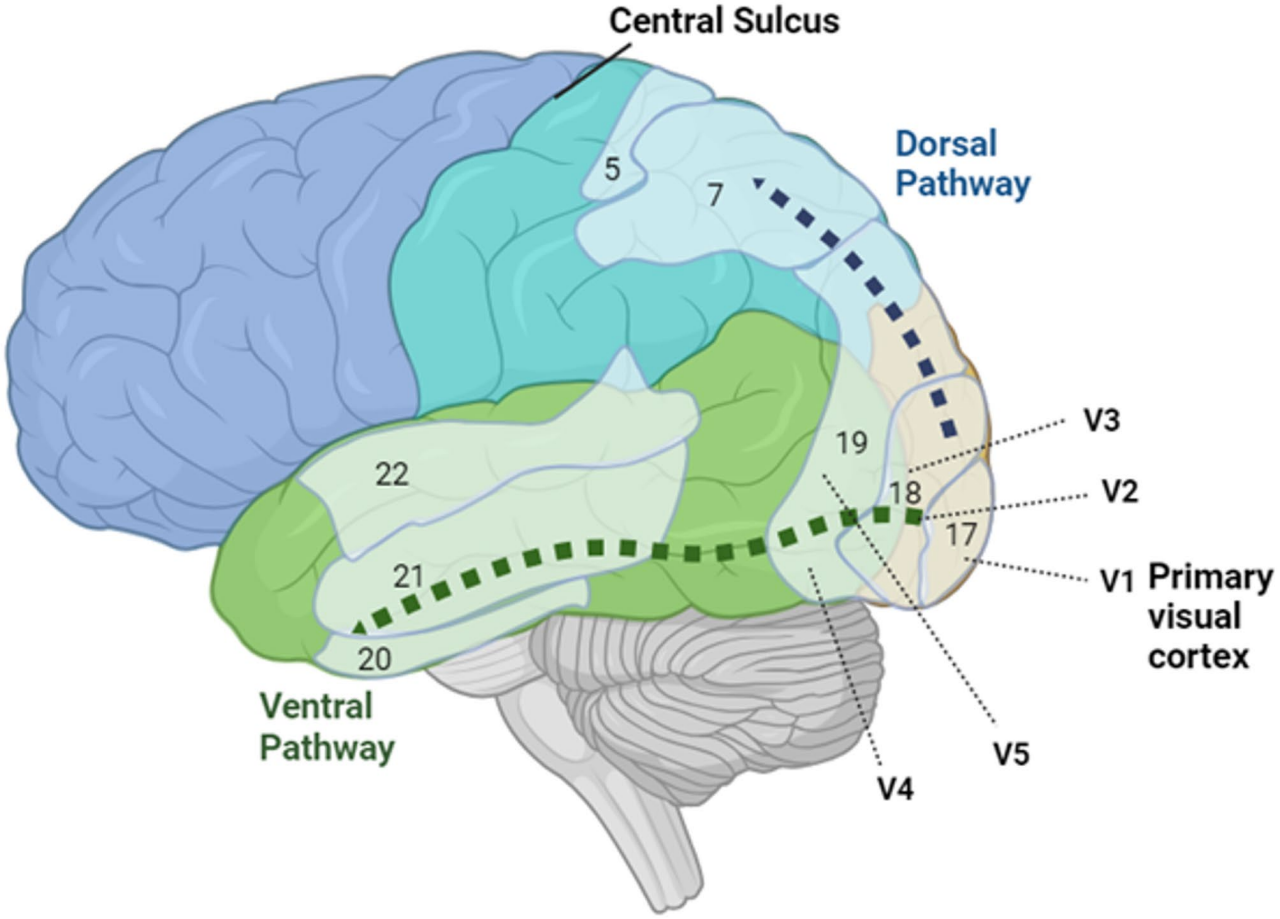
The ventral and dorsal paths of visual information processing. The pathways originate in the primary visual cortex (Brodmann area 17, or V1) and extend in two directions: ventrally through the occipital cortex to the inferior temporal lobe (green arrow) and dorsally to the posterior parietal cortex (blue arrow). The ventral pathway is responsible for identifying visual characteristics, whereas the dorsal pathway is associated with detecting object motion and spatial awareness. Figure created by Biorender.com

Signals from photoreceptors to the optic nerve are transmitted through the parietal and temporal lobes. The visual inputs that are transmitted from the optic nerve are first shared in the primary visual cortex/V1, which receives visual information from the LGN and processes simple components such as orientation and direction, and combines visual input to be passed on to other parts of the brain for further analysis and use [17]. From V1, the information is dispersed to several extra-striate cortical areas through the dorsal and ventral pathways. The dorsal pathway (Brodmann 21-occipital‒parietal pathway) is associated with detecting the motion of objects (speed selectiveness) and fast-tonic responses. The ventral pathway, including Brodmann areas 18 and 19 (occipital‒temporal pathway) and Brodmann areas 21 and 22 (inferotemporal pathway), is responsible for identifying visual information. This pathway processes visual input received from the thalamus, first arriving at V1 and then relaying to V3, V4, and V5 in the visual cortex. The input received through parvocellular cells is responsible for the slow-tonic response and object recognition. The V5 area is located between the parietal, frontal, and occipital lobes, and is thought to play a crucial role not only in motion perception but also shape perception, semantic processing, and attention [18, 19]. Patients with akinetopsia have lesions in the V5 area. As a result, perceptual asynchrony occurs between fast-moving and slow-moving objects. Patients can perceive and detect slow-moving objects but cannot perceive fast-moving objects [18]. Figure 2 summarizes the dorsal and ventral pathways of visual information processing.

### Lesion studies, brain imaging findings & disrupted neural mechanisms in akinetopsia

The etiology of this rare syndrome has been identified in a handful of patients with various conditions, such as cerebral infarctions in the occipitoparietal region that holds the V5 area. To date, all reported cases of akinetopsia have been associated with infarctions in the right hemisphere. There have been no reported instances of this condition resulting from localized left hemisphere infarctions. Maeda [5] reported a case of a 54-year-old man who was diagnosed with a fresh cerebral infarction in the right occipitoparietal region corresponding to area V5 following a traffic injury. During medical examinations, the patient reported that stationary objects appeared to move from front to back and seemed to grow larger. Clinicians have described optic flow, where objects move radially from the center to the periphery. Heutink et al. [15] described a 37-year-old female who experienced problems with perceiving visual motion following a stroke. Magnetic resonance imaging (MRI) revealed an ischemic infarction in the occipitotemporal region in the right hemisphere.

There are also reports of induced akinetopsic symptoms with the use of some medications and transcranial magnetic stimulation. Horton and Trobe [20] reported that selective serotonin reuptake inhibitors (SSRIs), such as nefazodone, caused selective impairment of pathways in the dorsal and ventral pathways of motion perception. Patients who were admitted to the hospitals due to nefazodone toxicity described their visual impairment as a “bizarre derangement”, as moving objects became a trial of “freeze-frame” images. Transcranial magnetic stimulation (TMS) of a tiny patch of the cortex (approximately 1 cm in diameter) corresponding in position to the area responsible for the experience of visual motion was found to selectively and irreversibly impair that perception [21].

**Fig. 3:**
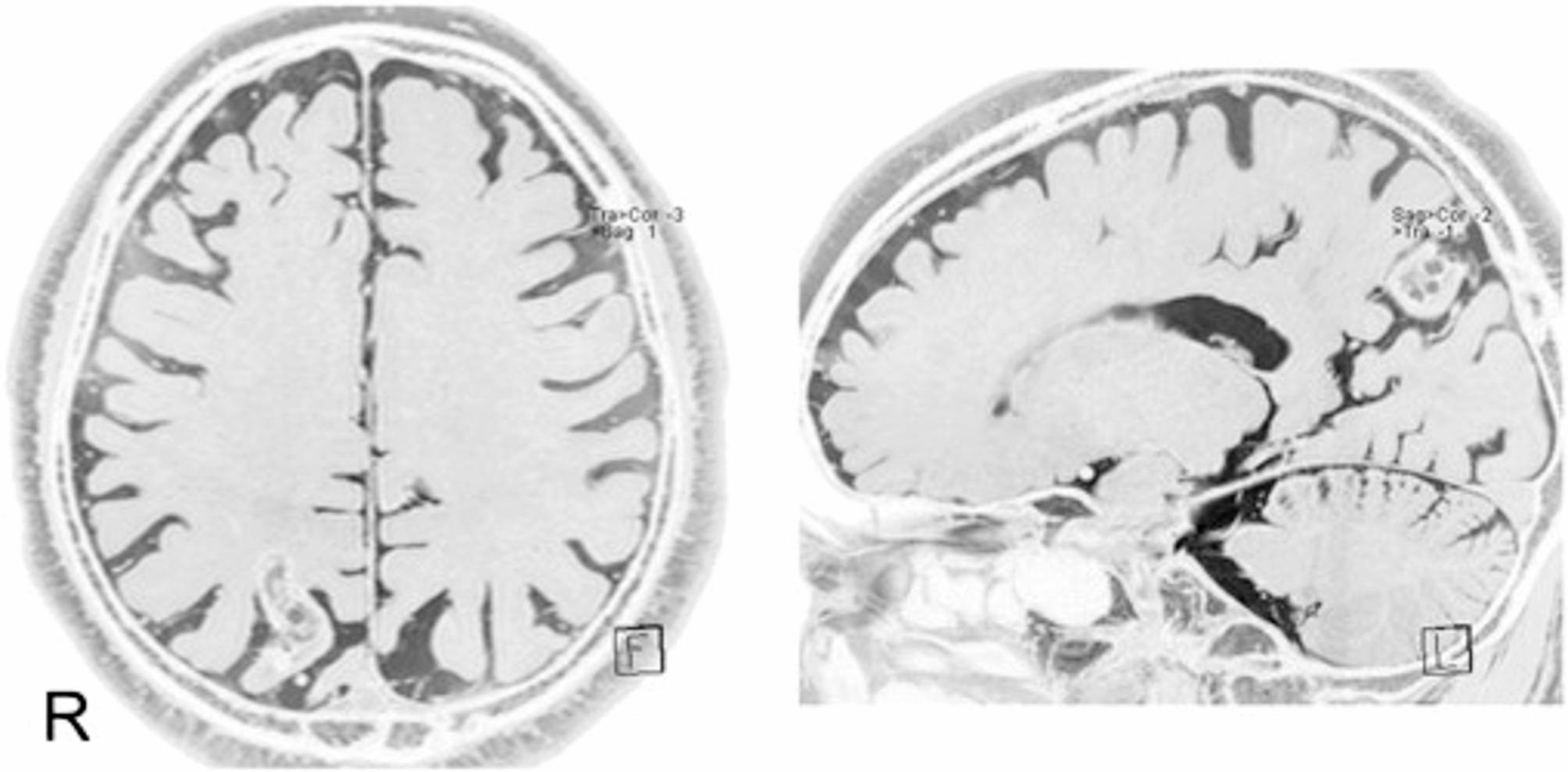
Magnetic resonance imaging. The lesion is visible in the right parietal lobe. Image from Sakurai et al., 2013 [14]. Akinetopsia as epileptic seizure. Epilepsy & Behavior Case Reports, Volume 1, Pages 74–76. © 2013 The Authors. Published by Elsevier Inc. Available under a Creative Commons Attribution 4.0 International License. (CC BY 4.0). No changes were made

Seizure disorders have also been implicated in patients developing akinetopsia. Maeda et al. [12] described several cases of akinetopsia accompanied by abnormal brainwave patterns in the right hemisphere. One 68-year-old woman reported experiencing an uncomfortable feeling that her vision would freeze, lose color, and resemble a black-and-white photograph. This symptom had been recurring daily. Electroencephalogram (EEG) recordings revealed abnormal brain wave patterns in the right hemisphere, whereas single photon emission computed tomography (SPECT) scans revealed increased blood flow in the right frontotemporal region and decreased blood flow in the bilateral occipital regions. Before treatment, the patient manifested reduced blood flow in regions of the brain associated with motion and color perception, especially in the bilateral classical centers of motion (MT/V5) and color (ventral part of V4; V4v). One hypothesis is that epileptic impulses originating from the right frontotemporal region may travel backwards through the right ventral visual pathway and suppress the functioning of MT/V5 and V4v on the same side, resulting in a loss of motion and color perception. Maeda et al. [12] reported a case of epilepsy focused in the right frontotemporal region, as confirmed by EEG and SPECT results, that demonstrated full-field akinetopsia with achromatopsia. Sakurai et al. [14] described another case of akinetopsia symptoms concurrent with focal epileptic seizures in the right temporal and parietal cortices, including the MT/V5 area (Fig. 3). The remote inhibition of the bilateral MT/V5 and V4 via the right ventral visual pathway and callosal connection could account for this rare symptom.

Several cases of akinetopsia developing from neurodegeneration and cancer have been reported. Tsai & Mendez [22] reported a case of a 61-year-old patient with Alzheimer’s disease (AD) who complained of localization in the left visual field and the sensation of double or multiple images when objects moved in the left direction but not while moving his eyes. Upon neurological examination, left hemi spatial neglect was diagnosed, and MRI revealed atrophy, especially in the parietal regions. Pelak & Hoyt [13] described a 70-year-old patient diagnosed with AD whose MRI showed bilateral posterior atrophy at the level of the occipito-temporo-parietal junction. They also [13] described a patient who developed akinetopsia following traumatic brain injury (TBI), resulting in posterior cortical atrophy; however, the specifics of this atrophy were not described in detail [13]. More recently, Viscardi et al. [2] provided the first case report of akinetopsia associated with brain metastases, indicating that this rare disorder is not limited to primary brain lesions or atrophy but can also be a symptom of secondary conditions such as brain metastases. These cases broaden our understanding of the potential causes of akinetopsia and highlight the importance of comprehensive neurological assessment in patients presenting with visual disturbances.

## Discussion

Akinetopsia, also known as motion blindness, is an infrequently diagnosed neurological condition that impairs the ability of the brain to process visual motion. First identified in the medical literature in 1911, akinetopsia has been documented in only a small number of cases since its initial recognition. The subjective experiences of patients with akinetopsia vividly depict the challenges and frustrations associated with this condition. One patient described feeling disoriented as “people were suddenly here or there but I have not seen them moving” [8]. Another patient “felt uncomfortable walking” because of difficulties in maintaining safe posture [23]. Others reported frequently bumping into moving individuals and an inability to catch moving objects [10, 23].

Akinetopsia has been associated with a variety of conditions, most notably with bilateral lesions to the occipital lobe affecting area V5. Cases involving bilateral occipital stroke and cerebral infarction provide staunch support for the role of area V5 in motion perception [11, 24]. Patients with neurodegenerative diseases such as Alzheimer’s disease (AD) can also experience akinetopsia relative to the degree of atrophy in their brain [13, 20]. These cases highlight the potential for akinetopsia to manifest as a symptom of various neurological conditions and the need for further research in the development of rigorous diagnostic tests for akinetopsia in tandem with neuroimaging. Akinetopsia can also be temporarily induced, as demonstrated in studies that used transcranial magnetic stimulation (TMS) or the use of certain medications, including nefazodone [20, 21]. When akinetopsia is induced by medication, clinicians can often mitigate the effects of the disorder by discontinuing the problematic drug. In cases of epilepsy, such as the one described by Sakurai et al. [14], the accompanying akinotopsic symptoms were completely suppressed with the use of antiepileptic medication. Regrettably, the cases of akinetopsia that were attributed to cortical lesions or neurodegenerative disease were irreversible. Only in the case described by Maeda [5] was akinetopsia resolved following a stroke because the infarction was still fresh and treatable with an antiplatelet agent. Future research can explore ways to diagnose akinetopsia in conjunction with imaging to better detect such infarctions in due time. In tandem, preserved motion perception following cortical lesions to visual pathways can provide a deeper understanding of motion perception pathways in the brain. For example, although cases of congenital V5 lesions have not been reported, Tinelli et al. [25] investigated visual perception in children with congenital versus acquired postnatal brain lesions. They found that children with congenital brain lesions to area V1 displayed preserved but unconscious visual motor perception in the blind hemifield, whereas children with lesions acquired during childhood did not, attributing this to neuroplasticity. Such cases can not only provide deeper understanding of visual pathways and roles of distinct areas like V5 but also emphasize the importance of early intervention and the brain’s potential for cortical reorganization.

Despite being a rare neurological disorder, further research on akinetopsia can provide valuable insights into the normal functioning of the visual system. Since patients with akinetopsia seem aware of their condition [13], it would be valuable to investigate the impact of this condition on patients’ quality of life to develop effective coping strategies. Longitudinal studies on patients with medication-induced akinetopsia could help us understand if and how the condition might resolve over time once the medication is discontinued. Future studies that explore the specific neural mechanisms of motion perception and how they are disrupted in akinetopsia can expand our understanding of the intricate neural mechanisms and pathways implicated in visual perception, thereby informing theories and models of standard visual processing. Table 1 synthesizes the etiology of akinetopsia reported in the literature.

**Table 1:** Summary of the etiology of akinetopsia with key findings. MRI = magnetic resonance imaging; eeg = electroencephalogram; SPECT = single-photon emission computed tomography; ct = computerized tomography; ad = alzheimer’s disease; hiv = human immunodeficiency virus

Reference	Etiology	Hemisphere affected	Primary symptoms	Secondary symptoms	Imaging/ Diagnostics	Outcome
[8, 10]	Stroke	Bilateral	Motion blindness, difficulty movement exceeding 10 dg/s	Headaches, vertigo, nausea, bilateral papilloedema	MRI, EEG, CT	Permanent deficits
[9]	Stroke	Bilateral	Poor motion detection and speed discrimination	Neurocognitive deficits	CT, MRI	Permanent deficits
[15]	Stroke	Bilateral	Impaired motion perception exceeding 9 deg/s and wagon wheel illusion	Nausea, dizziness	MRI	Unreported
[5]	Stroke	Right	Objects appeared to move/grow	None	MRI	Resolved with antiplatelet agent
[20]	Medication (Nefazodone)	Unspecified	Freeze-frame visual experience (“visual trails”)	HIV	MRI	Reversible following reducing/eliminating medication
[20]	Medication (Nefazodone)	Unspecified	Freeze-frame visual experience (“visual trails”)	None	Clinical observation	Reversible following reducing/eliminating medication
[12]	Epilepsy	Right	Freeze-frame visual experience, achromatopsia	Diabetes	MRI, EEG, SPECT	Resolved with carbamazepine
[22]	AD	Bilateral	Motion blindness in left visual field, poor saccadic pursuit	Double vision, memory decline, cataract	MRI	Progressive
[22]	AD	Unspecified	Motion blindness in leftward direction	Dressing apraxia, memory decline, depression, polyarthritis	MRI	Progressive
[2]	Tumor metastases	Multiple regions	Motion blindness. Impaired visually guided eye and finger movements, impaired depth perception	Asthenia, visual hallucinations, night terrors, decline in temporal orientation	MRI	Progressive with cancer

## Data Availability

Data sharing does not apply to this article, as no datasets were generated or analysed during the current study.

## Abbreviations

LGN: Lateral geniculate nucleus
EEG: Electroencephalogram
SPECT: Single photon emission computed tomography
AD: Alzheimer’s disease
TMS: Transcranial magnetic stimulation
BOLD: Blood oxygen level-dependant
MRI: Magnetic resonance imaging
SSRI: Selective serotonin reuptake inhibitors
